# Health care transitions for persons living with dementia and their caregivers

**DOI:** 10.1186/s12877-021-02235-5

**Published:** 2021-04-29

**Authors:** Jessica Ashbourne, Veronique Boscart, Samantha Meyer, Catherine E. Tong, Paul Stolee

**Affiliations:** 1 School of Public Health and Health Systems, University of Waterloo, 200 University Avenue West, Waterloo, ON N2L 3G1 Canada; 2 School of Health and Life Sciences, Conestoga College Institute of Technology and Advanced Learning, Kitchener, Ontario N2G 4M4 Canada

**Keywords:** Dementia, Health care system navigation, Health care transitions, Care coordination, Constructivist grounded theory

## Abstract

**Background:**

Persons with dementia are likely to require care from various health care providers in multiple care settings, necessitating navigation through an often-fragmented care system. This study aimed to create a better understanding of care transition experiences from the perspectives of persons living with dementia and their caregivers in Ontario, Canada, through the development of a theoretical framework.

**Methods:**

Constructivist grounded theory guided the study. Seventeen individual caregiver interviews, and 12 dyad interviews including persons with dementia and their caregivers, were recorded and transcribed verbatim. The data were coded using NVivo 10 software; analysis occurred iteratively until saturation was reached.

**Results:**

A theoretical framework outlining the context, processes, and influencing factors of care transitions was developed and refined. Gaining an in-depth understanding of the complex care transitions of individuals with dementia and their caregivers is an important step in improving the quality of care and life for this population.

**Conclusion:**

The framework developed in this study provides a focal point for efforts to improve the health care transitions of persons living with dementia.

**Supplementary Information:**

The online version contains supplementary material available at 10.1186/s12877-021-02235-5.

## Background

The term ‘dementia’ refers to a number of disorders resulting in a progressive decline in cognition [1]. Symptoms of memory loss and communication difficulty often precipitate dependence in activities of daily living (ADLs) with consequences for quality of life [1–5]. Dementia is often complicated by comorbid conditions and polypharmacy, necessitating care by multiple providers in multiple care settings [6]. Older adults with dementia use health care services more frequently than older adults without cognitive impairment [7].

Approximately 1.5% of the Canadian population has a diagnosis of dementia, and this proportion will nearly double over the next 30 years [1]. This growth will be accompanied by increases in economic consequences, system constraints related to demand for long-term care beds and community services, and an increase in unpaid informal caregiving hours from 231 million to 756 million hours per year [1]. There is thus an urgent need for effective coordination of health care and community support services for individuals living with dementia and their informal caregivers [8].

The nature of dementia frequently requires individuals and their caregivers to contact multiple health care sectors, and to experience transitional periods in which they need to navigate through an often-fragmented system [6, 7, 9–11]. A health care transition can be defined as “the movement patients make between health care practitioners and settings as their condition and care needs change during the course of a chronic or acute illness” [12]. The care transition process is one of increased vulnerability and risk, due to the potential for inadequate transfers of information, medication errors, and other adverse events impacting patient safety, care quality, and outcomes [5, 13–15]. The dementia experience exists amid current system- and organizational-level inadequacies in care coordination and continuity; overlaps and gaps in health care services further complicate transitions [11, 16–18]. Transitional care is a concept that has been previously described as “a set of actions designed to ensure the coordination and continuity of health care as patients transfer between different locations or different levels of care within the same location” [19].

Although system navigation and transitional care for persons with dementia have been recognized as priorities for policy [1], research [20], and performance improvement [21], there remains a lack of understanding of how these challenges might best be addressed. Most research on care transitions has overlooked older adults living with dementia [22]. In fact, a review of studies of transitional care programs [23] found that some studies purposefully excluded persons with dementia. Literature on transitions has largely focused on specific transitions (e.g., hospital-to-home) [18, 24, 25]. This trend is echoed in recent studies exploring transitions for persons with dementia [26–29]. For example, Gilmore-Bykovskyi and colleagues [26] obtained nurses’ perspectives on transitions for persons living with dementia from hospitals to skilled-nursing facilities. Therefore, additional research on care transitions from a cross-system perspective is warranted.

By focusing on cross-system transitions, this study respects the inherent complexity of the health care system. Understanding how health care in Ontario is organized can provide some insight into the system through which participants navigated. Health care in Ontario is governed by the Ministry of Health and Long-Term Care (MOHLTC). The MOHLTC divides health care spending into different sectors of operating expenses. Physicians are funded through the Ontario Health Insurance Program (OHIP), which consists of a portion of MOHLTC funding. As of 2017, most individuals (90.8%) in the province had access to a family doctor or other primary care provider [30]. The role of the family physician is to provide comprehensive medical care to individuals at all stages of life or disease in various health care settings. They are seen as a central hub for an individual’s care, acting as a gatekeeper for access to and coordination of additional health services, including referral to specialists [31]. In Ontario, regionalized health organizations provide further infrastructure to support planning and coordination of health services.^1^

Health care system improvements for persons living with dementia could be facilitated by a theoretical framework based on an in-depth understanding of how persons living with dementia and their caregivers experience transitions across the care continuum. The objective of this study was to explore the care transition experiences of individuals living with dementia and their caregivers and to use these perspectives to develop a theoretical framework outlining the factors affecting health care transitions.

## Methods

### Study design

Constructivist grounded theory methods [32] were used to fulfill the study objectives. In line with social constructivism, multiple realities and individual values were acknowledged and respected, and knowledge was co-constructed by researchers and participants. Memos and journaling helped maintain reflexivity [32, 33].

### Study participants

Recognizing the present and growing importance of informal caregivers for persons living with dementia [34, 35] both persons living with dementia and their caregivers were included in the study. Caregivers were also able to provide information about the transitions of those with dementia who could no longer communicate effectively. Eligible participants were individuals who: a) had a self-reported diagnosis of dementia or a were a caregiver for someone with a diagnosis, and b) had undergone a health care system transition since time of diagnosis. There were no explicit exclusion criteria based on dementia diagnosis or stage, so as not to risk the omission of important voices, as has been the case previously [23, 36, 37]. Care transitions were also defined broadly; participants must have moved between more than one health care provider or health care setting.

### Recruitment and study setting

Eight Alzheimer Society of Ontario chapters and one Family Health Team were approached via e-mail to help identify persons with dementia and their caregivers willing to participate. This yielded a participant pool of primarily community-dwelling persons with dementia and their caregivers. All interviews were conducted in the community setting; however, some of the individual interviews with caregivers represented persons with dementia who had transitioned into institutionalized settings, such as long-term care.

### Data collection

Preferences for individual or dyad interviews (including a person with dementia and an informal caregiver) were respected [38, 39]. All persons with dementia who were recruited preferred to be interviewed in the presence of an informal caregiver; some caregivers were also interviewed individually. Preferences for interview location were also respected. All participants chose to be interviewed in their own homes. Each participant was given an information letter, which was reviewed verbally. Transitions were described to participants as periods of time during which they moved from one health care setting to another or from one provider to another within the health care system. The researchers determined whether persons with dementia were able to provide consent by ascertaining whether the participants understood the nature of the research, appreciated the consequences of participation and understood alternative choices. Each person interviewed was able to provide his or her own consent. All participants who began the study continued until the end.

A background information form was completed using verbal information from participants; this provided data for sample description. Open-ended questions were used to obtain stories about care transition experiences. All questions were directed to persons with dementia first. If they did not answer, their caregivers were given the opportunity to provide details. Participants were first asked to identify the services, support and care that they had received since the dementia diagnosis. They were subsequently asked to describe a time during which they moved from one person or setting in the health care system to another person or setting. Further sample interview questions are summarized in Table 1 and the full interview guide is included in Supplementary File 1. With consent of the participants, interviews were audio-recorded. The interviews lasted approximately 1 h each.

**Table 1:** Sample interview guide questions and prompts

**Sample Interview Guide Questions & Prompts**	
**Can you tell me about time during which you moved from one person or setting in the health care system to another person or setting?** *Give examples if necessary* a. In what setting did you begin your experience? b. What care setting did you transition to?	
*Optional Prompts:* *Can you walk me through what happened? What aspects of your transition from X to X went well/could have been improved?* *Do you think that your diagnosis of dementia influenced this experience (if yes, in what way?)*	
**Was any organization or service helpful during or after your transition? What did X help you with? How has it been helpful?**	
**Can you tell me about any instructions that you received, if any, from the care providers about how you could manage your condition(s) on your own?** *Follow-up:* **Were these instructions important to your experience moving between providers/settings? Why/why not?**	
**Can you tell me anything about the communication that you observed or were aware of between the care providers that were involved in your care?** *Follow-up:* **Was this communication important to your** **experience moving between providers/settings? Why/why not?**	

Interviews were conducted by the lead author, a Master’s-level trainee with prior qualitative research training, over a seven-month period; verbatim transcriptions completed shortly after each interview allowed for simultaneous data collection and analysis.

### Data analysis

Following the analytic steps outlined by both Glaser [40] and Charmaz [41], we engaged in two stages of coding, after an initial read-through of the transcripts: first, open coding, and second, focused coding. Per Charmaz [32], theoretical coding was integrated throughout. The coding was completed by two data coders and the results were reviewed with a third analyst. In open coding, data were closely examined using line-by-line and incident-by-incident coding. Short, action-focused codes, or gerunds, were assigned to each small piece of data (for example: ‘maintaining a social life’, ‘seeing the future in others’ experiences’, ‘learning caregiver strategies’, ‘being prepared’, ‘having multiple providers in the room’). A constant comparative method, during which segments within and between interviews were compared throughout the data collection and analysis processes, was employed [32]. Guided by Charmaz [32], data were compared to other data throughout the entire coding process to elucidate similarities and differences. Observations and ideas that arose from the data were noted in memos [42]. For example, one memo noted how participants described their transition experiences in relation to others’ experiences. Comparative methods were used to identify and compare incidents in the data wherein participants spoke about others’ experiences. In focused coding, the most frequent or significant codes from the initial coding were synthesized from the data into a more coherent story [32]. The initial codes were grouped into categories and subcategories based on their similarities and differences, and relationships were suggested using memos and diagrams. The categories were refined and developed using theoretical sampling, a process by which preliminary categories are further developed by directing subsequent interviews. Charmaz states, “theoretical sampling can entail studying documents, conducting observations, or participating in new social worlds as well as interviewing or reinterviewing with a focus on your theoretical categories.” Given that it was difficult to choose new participants based on the emerging categories, probing questions were used to delve into specific aspects of experiences and further refine categories [32, 33]. For example, when people described their transitions, they often spoke about the events leading up to the transition. To further refine this category, participants were probed to describe how each transition experience began. This led to the refinement of the subcategory of influencing factors titled ‘catalysts’ in the results.

Data were collected and analysed until the authors believed theoretical saturation was reached; in practice, theoretical saturation was determined by noting a point in the coding process at which no new codes were required to explain the meanings behind participants’ stories [43]. After no new codes were required, an additional five interviews were conducted to confirm saturation. The researchers conducted member checks with participants by telephone; thirteen participants provided feedback.

### Theoretical framework development

When theoretical saturation was reached and confirmed, a framework was developed and refined to describe care transitions from the perspectives of persons with dementia and their caregivers and to fulfill the study’s primary objective. Since the study has been situated within a constructivist framework, the resulting theory is interpretive [32]. Diagramming was used throughout the data collection and analysis process to visually represent emerging categories and relationships; additional information about the diagramming and analysis can be found in [blinded for review].

### Sample description

Twenty-nine interviews were conducted in 15 cities or towns and three health regions (then organized as Local Health Integration Networks) within Ontario, Canada; this included twelve dyad interviews and seventeen caregiver-only interviews. Participants characteristics are summarized in Table 2. Participants presented with a range of self-reported dementia diagnoses; in order of frequency these were: Alzheimer’s Disease, Vascular Dementia, Mixed Dementia, Mild Cognitive Impairment, Fronto-temporal Dementia, Parkinson’s Dementia, and Dementia with Lewy Bodies. Over one quarter of participants reported a non-specified or unclear diagnosis. Individuals received their dementia diagnoses from a variety of providers including geriatricians, primary care physicians, physicians at memory clinics, neurologists, and one doctor in a care home. Per the eligibility criteria, all participants had experienced a transition with the health care system after a diagnosis of dementia. Transitions experienced by participants included those between primary care providers, specialists, memory clinics, hospital, respite care, long-term care home, and home care providers.

**Table 2:** Participant characteristics

Participant Characteristics (***N*** = 41, in 12 dyad and 17 individual interviews; ***n*** = 29 for persons with dementia who were represented in the interviews*)	
	Mean (min, max)
**Age**	
Persons with dementia	78 (min: 58, max: 94)
Caregivers	69 (min: 56, max: 80)
	**N (%)**
**Participant Type**	
Persons with dementia	12 (29.3)
Caregivers	29 (70.7)
**Gender**	
Persons with dementia- Women	6 (20.7)
Persons with dementia- Men	23 (79.3)
Caregivers- Women	25 (86.2)
Caregivers- Men	4 (13.8)
**Relationship of Caregivers to Person with Dementia**	
Son	1 (3.4)
Husband	3 (10.4)
Daughter	4 (13.8)
Wife	21 (72.4)
**Types of Dementia Diagnosed**	
Lewy Bodies	1 (3.4)
Parkinson’s	1 (3.4)
Fronto-temporal	2 (6.9)
Mild Cognitive Impairment	2 (6.9)
Mixed	3 (10.4)
Vascular	4 (13.8)
Alzheimer’s	8 (27.6)
Non-specified	8 (27.6)
**Number of chronic conditions of persons with dementia**	
0–1	15 (51.7)
2–3	5 (17.2)
4–5	5 (17.2)
6+	4 (13.8)

*﻿While only 12 persons with dementia were directly interviewed, *n* = 29 refers to the persons with dementia who were either interviewed directly in a dyad interview AND who were spoken of/for in the caregiver interviews

This study received ethics clearance through the [blinded for review] Office of Research Ethics.

## Results

### Theoretical framework

**Fig. 1:**
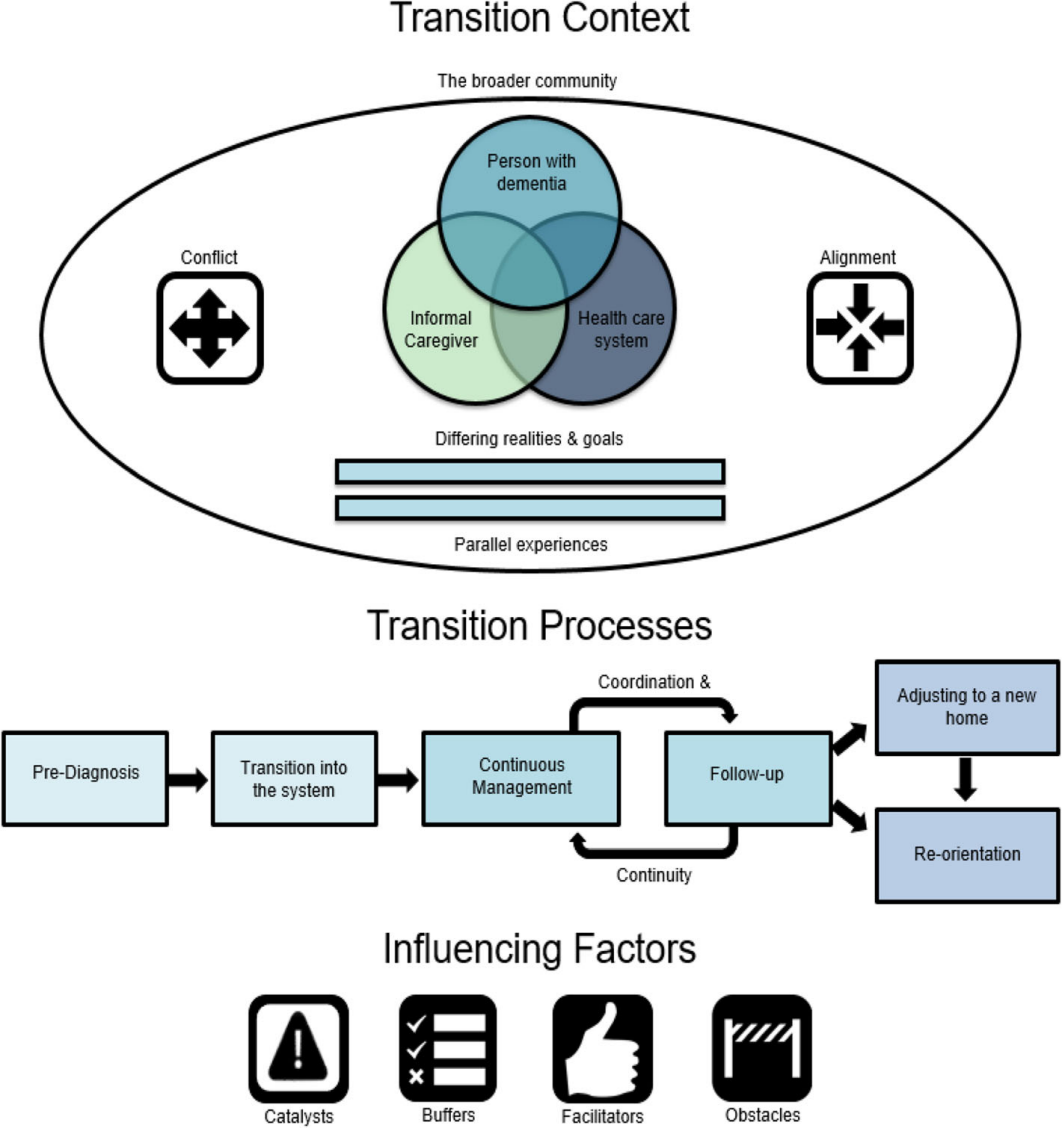
A three-part theoretical framework outlining the context, processes and influencing factors of care transitions from the perspectives of individuals with dementia and their caregivers

The final representation of the emergent framework of dementia-specific transitions is depicted in Fig. 1. Key themes that emerged from the data were organized into three categories: transition context, transition processes, and influencing factors. Identifying important contextual elements respected the fact that transitions did not occur within a vacuum; they were situated within communities and influenced by the perceptions and aims of participating parties. Subcategories of transition context reflected differing realities and goals among those involved in the transitions, the parallel experiences of other individuals undergoing similar transitions, and the broader community. Rather than discrete instances of movement from setting to setting, transitions were experienced as continuous and linked to phases of the dementia journey. Subcategories of transition processes described by participants included the transition into dementia care, a continuous process of management and follow-up, and adjustment to a new home and reorientation. Four subcategories of influencing factors were identified within these processes: catalysts, buffers, facilitators, and obstacles.

### Transition context

#### Differing realities and goals: in conflict and alignment

*Conflicting realities* often led to communication difficulties during transitions. One caregiver, 57, explained how her husband’s view of reality did not align with hers in saying: *“…his opinion of what he is capable of and reality is not always the same.”* She continued on to express her concerns for safety related to such disparities in perception, indicating that her husband’s descriptions of his own abilities made him seem in need of less support than she deemed necessary. A similar concern was presented by a daughter, 56, in caring for her father, 87. She stated: *“…at one point he was on 20 to 22 prescription drugs per day, but you’d ask him, and he’d say, ‘No I don’t take pills.’”* These caregivers expressed worry that the health care system was not getting the information required to properly assess the transition needs of the person with dementia. Thus, conflicting realities may impact the appropriateness of recommendations (e.g., long term care placement) and referrals (e.g., to specialists).

Similarly, *conflicting goals* were indicated to have caused problems during transitions when participants felt that their views were not respected in the health care team. Sometimes, system goals directly opposed and “outranked” the goals of persons with dementia and their caregivers. One wife and caregiver, 68, described her conflict with system-level goals in a story about trying to access home care support:

*Trying to book these events…[the coordinator’s] goals were different than mine. Mine were to make [my husband] happy with the people that came, to try and make it as few new faces as possible… Her goal was to have you be specific about the exact times that you would want those PSWs (personal support workers, a.k.a. nursing aides), and to have it a regular weekly booking.*

In this scenario, system constraints were prioritized over her wishes, but this was not always the case: *“They didn’t have rules. They had ways of, rather than a rule, they had a way of giving me support. They found a way”* [wife (caregiver), 78]. When the system *did* accommodate the person living with dementia and their caregiver, they had more positive experiences.

#### The broader community

Many transitions occurred while persons with dementia were living at home, and thus their communities were considered an important part of their context. Knowledgeable and supportive communities helped individuals live well at home by supporting caregivers and sharing responsibility: *“It made my life easier. I didn’t have to be the…Helicopter mother…Or wife…When he was out walking I knew I had eyes on the street”* (wife/caregiver, 65)*.*

Community support was greatly appreciated by both caregivers and persons with dementia; it is thus important that providers are aware of a family’s supports when assessing them. One caregiver, 77, described how a provider inquired about her community support during her husband’s transition out of hospital: *“She noticed how he was and she said to me, ‘what help do you have at home?’ And I said, ‘I don’t have any.’ And she said, ‘you just take him right upstairs and say you’re not going home until you get help.’”* Despite taking the initiative to ask about support, the onus was placed on the caregiver to find help, indicating that more could potentially be done by the provider. By understanding the available community and family support systems, providers can better understand the context of transitions and help facilitate strong community support systems. These support systems may influence the need for transitions; for example, caregivers who have more community support may delay a transition to a formal care environment, such as long-term care.

#### Parallel experiences

The final aspect of transition context was labeled *parallel experiences*, which are shaped by others’ anecdotes. Rather than having a direct influence on care, parallel experiences provide context because they affect perceptions of care experiences. Hearing about others’ experiences further along in the dementia journey allowed participants to better understand what might happen to them: *“The caregivers that we have, there’s different stages. So you kind of get an understanding of what might be down the road. Which is very nice to get feedback from them”* (wife/caregiver, 67). People used their interactions with others to learn about potential future experiences, which may colour perceptions of impending transitions.

Individuals understood their own experiences in relation to anecdotes heard from others. When speaking of others’ negative experiences, participants reported feeling comparatively fortunate or lucky. For example, one caregiver noted: *“We have been very lucky…‘cause I’ve heard some horror stories.”* One daughter and caregiver, 61, expressed a similar sentiment: *“I was surprised when I do meet people and they seem to have all these challenges. We were just very fortunate… I’ve heard horror stories. But never from my point of view.”* Less negative experiences than those reported in others’ stories were viewed in a more positive light in contrast. These parallel experiences are an important finding, as they indicate how peer supports can help individuals prepare for and cope with potentially difficult transitions.

### Transition processes

#### Pre-diagnosis and transition into the system

Participants relayed stories about several transition points during their care, starting with diagnosis and transition into the care system. System entry was described as either gradual or sudden, each presenting different issues. For some, a slow progression eased the transition into the system; they knew it was coming, so they had been mentally preparing for years. For others, this gradual transition represented a failure of the health care system to address their needs in a timely fashion. A wife and caregiver, 64, noted: *“…it took… a long time to convince [the family doctor] that [my husband] had memory issues.”*

Some participants described feeling like they had been waiting for a crisis to occur. When crises did occur, people were “…propelled into the care that [they] needed” (wife and caregiver, 57), suggesting a sudden entry into the system. Crises were often said to follow a period of waiting for a diagnosis; however, in a few cases the diagnosis was also sudden and a surprise. For one wife and caregiver, 72, “…it came as a shock when [my husband] was diagnosed.” Participants’ stories also suggested that the perceptions of initiation of care are influenced by how they receive a diagnosis. This initial transition sets the stage for the rest of the journey through the health care system, as a diagnosis can provide an entry to support services and treatment. For example, one wife and caregiver, 64, stated: years. *“[My husband]* *was diagnosed on a Friday. I called them on Monday, and by Thursday there* *was a…coordinator at our house…she has been by our side every step for the last six years.”*

#### Continuous management and follow-up aided by coordination and continuity efforts

When responding to questions about the types to transitions they had experienced, participants described transitions in care after the initial diagnosis as a process of continuous management and follow-up. One wife and caregiver, 74, noted:

*I mean our transitions have…from one thing it seems to be…on-going…I don’t think that’s ever going to be at a standstill. Because of age and because of …some of the medical diagnoses…it’s always going to be a continuous transition.*

Regarding his mother’s care experiences, one son (caregiver), 56, suggested that *“it all flows together.”* An individual with dementia remarked that his care consisted of *“just visits to the doctor’s office, or the hospital, whatever comes first.”* Another caregiver, 74, felt that her husband’s transitions were so intimately linked that she was *“almost barely aware of the transitions.”* Many care transitions may occur during the course of a journey with dementia; however, these are often not considered discrete instances of movement from provider to provider. They are perceived as individuals’ reactions to changes in the dementia symptoms, in their needs, and in their lives.

During continuous transitions and adaptations, participants desired regular follow-up by those involved in their care: *“I think that they need to be the ones that are following up, and checking maybe every six months to see where things are at”* [wife(caregiver), 70]. Persons with dementia and their caregivers often required ongoing care from several providers; therefore, efforts to ensure coordination and continuity of care helped connect the transition processes.

Important aspects of coordination included clear referrals to appropriate services, professional collaboration, and information transfer. There was a perceived need for providers to be aware of and refer to each other. Beyond that, participants believed that providers should work together to meet their needs. One caregiver, 67, described a lack of collaboration among providers in her area: *“Well, a lot of them up there… they’re all, on their own… each one… so they got their own controls. I think it’s the biggest thing here.”* Further, a lack of information transfer contributed to deficits in provider collaboration. A wife and caregiver, 78, noted: *“We made sure ourselves. We didn’t just trust them, and it didn’t all just fall into place… They didn’t do it the way they said they would.”*

Regarding the goal of care continuity [44], one wife and caregiver, 80, explained, concerning her husband’s care: “*Having a doctor see [my husband] every six months… I know that someone has his journey on paper.”* Relatedly, participants indicated that maintaining consistency in providers was preferable: *“You need a little bit of continuity here with these poor people who don’t even know who they are themselves”* [wife(caregiver), 68]. Another wife and caregiver, 68, described how consistency made the assessment process easier: “*If you have somebody who’s been here three times before, she’s familiar with your file and she… didn’t have to start down here all over again.”*

#### Adjusting to a new home and reorientation

Although most care transitions were viewed as continuous, many participants indicated that moving to long-term care represented a distinct, difficult transition. One daughter and caregiver, 56, called it a *“big step”*, while a wife and caregiver, 77, noted that *“it’s no easy route.”* Caregiver feelings of loss of control contributed to its emotional significance: *“It’s also overwhelming to be in a position where you have to face the fact that you cannot do it, and you have to turn his care over to somebody else*” [wife (caregiver), 77]. When the father of a daughter and caregiver, 56, moved into long-term care, the environmental shift was a major stressor for him as well:

*He took a dive bomb as far as… understanding things, being able to… he had no idea he was in [city]. Like, ‘when are we going back home’… like he just dive bombed horribly and stayed that way for many, many days.*

Familiarization with the long-term care environment prior to the transition was suggested to ease the transition. One wife and caregiver, 72, suggested, *“there might be some advantage to having him in [respite care] there because as the thing progresses, he will be stabilized in a place that’s familiar.”* When transitions to long-term care went smoothly, persons with dementia began to adjust and consider the facility their new home. One husband and caregiver, 73, noted that positive experiences allowed his wife’s long-term care facility to *“become her home.”*

A progression of dementia symptoms and the move to long-term care marked the need for reorientation or finding a new normal. One wife and caregiver, 65, indicated that she had trouble knowing what to do when she no longer had the full-time job of taking care of her husband: *“What am I supposed to do with this time?”* Another wife and caregiver, 72, said she had trouble transitioning from doing things together to doing them alone: *“We always discuss this together. I’ve got to make a decision all by myself… I’ve heard that other people say that all of a sudden, ‘I’m just alone.’”* One husband, 64, described his transition into a new role and learning to move on without his wife: *“So now my transition is… the challenge for me transitioning as an individual, is how do I re-orient my life?”*

### Influencing factors

#### Catalysts

Participants described factors that led to their transitions. Precipitating crises included falls, overdoses, heart attacks, surgeries, and major behavioural problems. One caregiver, 71, revealed that her husband had a heart attack that worsened his dementia symptoms, thus, necessitating care outside of the home for a period of time: *“When he had his heart attack, that spiked his dementia, and he was really confused and didn’t know where he was and what day it was and I could not bring him home in that condition.”* Events and crises in the lives of caregivers also precipitated transitions. One wife and caregiver, 77, noted: *“So then it came to the point where I got a knee problem… So then I was booked for surgery. So then we decided that we’ll book him for the nursing home.”*

Not all transitions were preceded by major events or crises. Participants’ ability to manage at home changed over time, leading to different care requirements. One wife and caregiver, 57, noted a change in coping, which led her to have her husband assessed for home support: *“we were getting to the point that we weren’t coping very well, which is why I made the call.”* Similarly, another wife and caregiver, 72, began to have an increasingly difficult time with her husband’s eating problems, leading her to reach a tipping point: *“I thought, well, I’ll take him home, and tomorrow I’m going to call her and tell her I can’t do this anymore, I just, I’ve reached the end.”*

#### Buffers

Pre-transition proactivity and preparation can provide an emotional and physical buffer for care transitions. A husband and caregiver, 64, found that taking necessary steps and preparing for the future allowed him to become resilient to the transitions to come:

*If you’re able to step back and take steps like getting the powers of attorney, adjusting the household arrangements…you become much more resilient, and that resilience cushions you against… the next part of that transition.*

Participants reported having to advocate for their own care while moving throughout the system. One person with dementia, 73, stated, *“You have to be aggressive, and if you’re not an aggressive type person then you’ll miss out on those things. You’ll just go by.”* A wife and caregiver, 84, illustrated the connection between self-advocacy and the need for individuals to be educated and prepared for health care encounters: *“We’ve definitely got to be our own advocates when we go to the doctor’s. You’ve got to know a little bit about some stuff.”* People desired information about stages of dementia, future transitions, and system processes.

One wife and caregiver, 58, suggested that her knowledge provided an advantage: *“Knowing what the process is, and how lengthy it is, and knowing some of the little techniques to put yourself in a better advantage.”* By gaining a better understanding of dementia and the health care system, participants were able to prepare for future transitions:

*I knew I didn’t need anything at the time, but I just wanted to make sure that I had all of my research and homework done... When I do need assistance... I’m already in the system.* (wife and caregiver, 64)

#### Facilitators

Facilitators are factors that subjectively contributed to the success of transitions. Participants appreciated providers who were understanding and compassionate while also professional and skilled. Comments about provider attitudes often involved statements about providers’ professionalism and expertise. One wife and caregiver, 80, noted that her husband’s physician in the memory clinic was *“a very gentle man and he always has time for you,”* but also that “*he’s the one who calls the shots,”* suggesting she valued both his attitude and his expertise.

Participants recognized that the development of a working relationship with a provider required effective engagement. One participant with dementia, 92, described how his providers included him, along with his caregiver in decision-making: *“very cooperative in discussing things with us. Not saying you must do this.”* A wife and caregiver, 71, suggested that people with dementia should be given the right to participate in decisions: “*So for the few that really are able to choose, I think that they should let them.”*

Caregivers suggested that it was important that the health care system viewed persons with dementia in the same way they are viewed by their loved ones: as people with individual needs: *“They’re still people”* (wife and caregiver, 68); *“It’s important to see the person as the person instead of as the disease”* (wife and caregiver, 70). Furthermore, one wife and caregiver, 74, believed that the system should accommodate individual needs: *“I think that they should be able to look at that and…make it work for individual people.”* Another caregiver, 56, revealed that her father’s transition into long-term care was eased by the person-centered care he received: *“They make it their business to know what that person did in their life and what’s important to them.”*

#### Obstacles

Finally, transition obstacles are system, provider, or individual-level factors that hinder transitions. Transitions may be impeded by the complexity of the health care system and resulting confusion. Furthermore, the health care system is also constrained by factors such as time, money, and strict regulations.

There was a perceived need for increased knowledge and education of providers: *“I think that as time goes on it is an area where more training is going to be needed with all staff. Because more and more of this is coming”* (wife and caregiver, 65). Knowledge about rarer dementias was described as requiring improvement: “*I feel that they do have some training… but more so it seems to me that it’s more of an Alzheimer’s training”* (daughter and caregiver, 68).

Participants felt that their ability to successfully navigate the health care system was hindered by information or communication deficits. One wife and caregiver, 72, felt her providers were withholding information from her:

*So that’s what I mean by not always lying but just holding information in. And I got that feeling all the time that it was all private information and I had the bloody nerve thinking I should be entitled to stuff.*

When participants’ providers did not provide them with enough useful information, they felt they had to go looking for it themselves, which contributed to their stress: *“You have to go searching and that’s just one more thing in the day”* (wife and caregiver, 67). Participants emphatically noted that engagement of persons with dementia, and their caregivers, must begin with appropriate and clear communication.

## Discussion

It is clear from the diversity of information that arose from the interviews that care transitions in the context of dementia are complex. Given the significant heterogeneity of transition experiences discussed by participants, it became clear that transitional care cannot be fully described by a series of actions taken to ensure that an individual moves seamlessly from one setting in the health care system to another, as it has been classically defined [19]. Participants typically did not view transitions as discrete events that could be studied, assessed and/or improved without an understanding of both their personal journey and the broader health care system. The theoretical framework presented in the results section aims to distil a multifaceted topic while respecting its complexity.

Many of the features of existing transitional care models and interventions are consistent with the results of this study. Coleman et al’s [45] *Care Transitions Intervention* (CTI) is tailored to individual goals, indicating consideration of the multiplicity of goals in health care practice. Both the CTI and Naylor and Van Cleave’s [46] *Transitional Care Model* consider factors such as follow-up, professional collaboration, provider consistency, patient education, engagement, and person-centered care; all of which were also identified as important in the theoretical framework. That said, these interventions focus on specific transitions, typically a hospital discharge or acute event [45–47]. The broad perspective adopted for this study facilitated the development of a framework that elucidates aspects of several types of transitions while respecting the continuous nature of transition experiences. The theoretical framework presents a novel contribution to the literature, as it aims to avoid an over-simplification and does not assume a one-size-fits-all transitional care model.

Effective transitional care has the potential to reduce issues associated with fragmentation; however, reductionist thinking can be a significant barrier to the provision of care across the continuum. Complex systems theory has a natural application to the concept of transitions. Cilliers [48] noted: “A complex system is not constituted merely by the sum of its components, but also by the relationship between these components. In ‘cutting up’ a system, the analytical method destroys what it seeks to understand.” (p. 2). This study indicates that the complex nature of transitions must be respected; rather than reducing transitions to their individual components, transitions are viewed by both persons with dementia and their caregivers as continuous and are situated within a broader context. Despite being asked to describe specific instances of transition, participants tended toward describing their journey through the health care system more broadly. Although some specific transitions (i.e., the transition to long-term care) stood out as distinct and important to participants, separating the transition experience into discrete instances of movement in the health care system does not adequately explain their experiences. Rather than constricting individuals to view transitions through the lens of previous studies and health care system norms, this framework respects the reality presented by the participants.

Distinct from previous work on transitions for older persons [e.g., 45], our framework here urges both practitioners and researchers to think of health care transitions for persons with dementia not as events, but rather another aspect of their dementia journey [49]. The dementia journey is a complex one, often with multiple transitions, and which the caregiver and person with dementia may experience differently. Both Coleman’s [e.g., 45] and Hirschman’s [50] programs include family caregivers, but in these articles, patients and family caregivers are generally referred to together, without differentiation. Our framework also emphasizes the parallel experiences and differing realities and goals that occur within the context of dementia care. Misaligned priorities within dementia care have also been documented in Gilmore-Bykovskyi et al.’s [26] study. During transitions from hospital to skilled-nursing facilities, nursing staff commented on the impact that misalignment in system-level pressures and goals between these settings had on the transition process for persons with dementia [26]. Similarly, Richardson et al. [51], in a systematic review aiming to understand transition experiences of individuals with dementia from the perspectives of key stakeholders, found that the perspectives of individuals with dementia, their family members, and care providers did not always match. Hospital pressures led to rushed discharge practices and worse transition experiences. Involvement of families and adequate communication between stakeholder groups facilitated successful transitions [51]. By including the perspectives of caregivers and persons with dementia, our study further explored the idea of alignment in dementia care contexts. Varying realities and goals between caregivers and persons with dementia led to difficulties during transitions. Similar to Gilmore-Bykovsky and colleagues’ study [26], in this study wider health care system-level pressures and constraints at times conflicted with the care needs of persons with dementia and their caregivers. We hypothesize that differing priorities and realities may also be true with older adults who are not living with dementia, and this would be an important avenue of future transitions research. In other work of our group, we investigated care transitions of older individuals with hip fracture [18]; the resulting framework also differentiated family caregivers as a specific area of focus, while recognizing overlap with other domains.

Our framework also highlights that a dementia journey, and transitions embedded within, is significantly impacted by a range of catalysts (often acute events), buffers (e.g., proactive caregivers), facilitators (e.g., positive relationships with health care providers), and obstacles (e.g., lack of awareness and training). In order to appropriately intervene, and support persons on this journey and through numerous complex transitions, practitioners must be aware of the unique blend of catalysts, buffers, facilitators and obstacles that each person living with dementia faces. This observation, and the foundation of our model, is closely aligned with the principles of person-centered [52] and collaborative care [53].

Collaborative patient-centred practice, which “is designed to promote the active participation of each discipline in patient care” [53], can play an important role in addressing transition complexity. Encouraging collaboration across the care continuum rather than between discrete sets of providers can help to ensure that the provision of health care aligns with the way care is experienced. Persons with dementia and their caregivers have individual goals, needs, and perspectives that must be considered during care transitions. A commitment to respecting realities of individuals with dementia and adopting person-centered transitional care approaches can help ensure that system constraints do not overshadow individual needs. This could ultimately enhance quality of care for persons with dementia and limit the occurrence of adverse events [22]. Consistent with Fortinsky & Downs [49], we agree that future work on care transitions for persons with dementia must focus on improving the quality and experiences of care transitions, and not simply focus our efforts on delaying or avoiding transitions. Even with prevention and diagnostic efforts in place, persons living with dementia *will* experience transitions, and those charged with improving dementia care must plan accordingly [49]. Individual and family engagement in care can also help providers gain an understanding of the context of transitions, including the community and social support available to those navigating the system.

## Limitations and strengths

Limitations of this study included an overrepresentation of well-supported female caregivers and of males with dementia. Recruiting through Alzheimer Society Chapters and a Family Health Team may have yielded a sample more connected to community supports than an average Canadian with dementia. Furthermore, during interviews, caregivers spoke more than their family members with dementia. Despite addressing individuals with dementia directly, persons with dementia often referred to their caregivers. Finally, approximately one quarter of participants reported an unclear diagnosis, though all identified with the umbrella term of dementia. Whether it is related to a lack of clarity in terminology used by providers or poor understanding on the part of the persons with dementia or caregivers, this hazy experience with diagnosis reflects participant realities, which are considered paramount within a constructivist paradigm. In practice, specific dementia diagnoses are based on constellations of symptoms and cognitive testing; therefore, the diagnostic uncertainty within the umbrella of dementia observed in this study reflects the reality of many practitioners as well [54, 55]. Rather than limiting the generalizability of the study, the broad inclusion of persons who identify with a diagnosis of dementia supports the generalizability of the results to a larger population.

We did not conduct theoretical sampling for transition type because we found that participants often described multiple transitions, and their transition experiences were not always distinct. Based on these initial observations, we used probing questions to further explain the elements of transitions that arose during initial interviews rather than explicitly asking about or sampling for specific transitions. We felt that this approach respected the realities of the cross-system transitions experienced by those that were interviewed. The heterogeneity of types of transitions described by participants made it difficult to provide specific guidance on improving any one given transition (e.g., hospital to home or home to long-term care). However, this study does provide important information about how individuals perceive their health system navigation more generally. The results respect the realities of participants, who feel that their transitions are less distinct and more fluid than traditionally perceived and defined in health care systems research.

Criteria for evaluating grounded theory research outlined by Charmaz [32] were considered throughout the study design. Credibility was maintained through gathering rich data, transcribing interviews verbatim, and ensuring that initial codes remained close to participant wording. The study has met the criterion of originality, as it addresses a gap in the literature. Resonance with participants was ensured through member checks, wherein researchers presented results to participants for feedback. Nearly half of the participants (*n* = 13) engaged in member checks, and every individual agreed that the framework accurately and comprehensively reflected their health care transition experiences. As for the usefulness of the study, it has added a new perspective of cross-system care transitions from the perspectives of those with dementia and their caregivers. It is situated within the Ontario health care system; however, many of the elements deemed important by participants are likely more broadly generalizable.

## Conclusion

This study extends current knowledge to include an in-depth account of care transitions from the perspectives of individuals with dementia and their caregivers, who have often been excluded from related research. Obtaining the perspectives of individuals with dementia is not always easy, and their views do not always match those of their caregivers or their health care providers [56]. We chose to adopt a constructivist outlook that respected these differing realities and perspectives, an idea that was central to transition context.

It is important to incorporate the perspectives of persons with dementia and their caregivers into our knowledge of the dementia journey. Understanding the experiences of persons with dementia interacting with the health care system can help facilitate a more holistic understanding of care transitions, however, this study did not compare the care transitions of persons living with dementia to those of persons without dementia. Due to the intimate link between participant stories and the dementia experience, the voices of persons with dementia cannot be generalized to the voices of all other older adults. Care transitions were often associated with stages of their dementia journey; the diagnosis, for example, signified a transition into the system. Transitions were interrelated and continuous when participants were living at home; a transition into long-term care may be required when the dementia had progressed significantly [57].

The conceptualization of transitions developed in this study may be used to generate recommendations to improve dementia care across the continuum, in particular as local and national dementia strategies are presently being developed and implemented [58, 59]. Given the significance of transitions for safety and caregiver stress, improving care transitions may in turn improve the quality of care and quality of life for individuals living with dementia.

## Supplementary Information

**Additional file 1.** Interview Guide.

## Data Availability

The datasets used and/or analysed during the current study are available from the corresponding author on reasonable request.

## Abbreviation

ADLs: Activities of Daily living

## Footnotes

1 At the time of data collection for this study, these were the Local Health Integration Networks (LHINs); LHINS are currently evolving to a new organizational structure and identity.
