# Supplementary material for: Health care transitions for persons living with dementia and their caregivers
Source: BMC Geriatr. 2021 Apr 29;21:285. doi: 10.1186/s12877-021-02235-5 (PMC8086075; doi:10.1186/s12877-021-02235-5)
Supplement: Supplementary file 1 — Additional file 1. Interview Guide. [file 12877_2021_2235_MOESM1_ESM.docx]

**Health Care Transitions for Persons Living with Dementia and their Caregivers**

**Supplementary File 1. Interview Guide**

**Jessica Ashbourne, MSc,** School of Public Health and Health Systems, University of Waterloo, ON, Canada, N2L 1P3

**Veronique Boscart, PhD,** School of Public Health and Health Systems, University of Waterloo, Waterloo, Ontario, Canada, N2L 1P3. School of Health and Life Sciences, Conestoga College Institute of Technology and Advanced Learning, Kitchener, Ontario, Canada, N2G 4M4

**Samantha Meyer, PhD,** School of Public Health and Health Systems, University of Waterloo, ON, Canada, N2L 1P3

**Catherine E. Tong, PhD,** School of Public Health and Health Systems, University of Waterloo, ON, Canada, N2L 1P3

**Paul Stolee, PhD,** School of Public Health and Health Systems, University of Waterloo, ON, Canada, N2L 1P3

**Interview Guide**

CONTEXT QUESTIONS

1. Can you please tell me about the services, support and care that you have received since you have

been diagnosed with dementia? (*Give examples: specialist care, family physician, memory

clinic, CCAC and other community supports*)

2. Can you tell me about any experiences that you’ve had within the health care system that have

gone particularly well?

a. What was it that made this experience so positive?

b. Who was involved? What did they do to make the experience positive?

3. Can you tell me about any experiences that you’ve had with the health care system that did not

go so well?

a. What was it that made these experiences negative?

b. What could have been done to improve your experiences?

TRANSITION QUESTIONS

4. The idea of care transitions basically means moving from provider to provider or setting to setting

in the health care system.

a. Can you tell me about what has gone well for you while moving or navigating through

the health care system?

i. What has made your transitions go smoothly?

ii. Who was involved?

iii. Are there any specific organizations?

b. Can you tell me about what has not gone so well for you while moving or navigating

through the health care system?

i. What could have been improved to make your movement in the health care

system easier?

5. Do you think that having dementia (or the person you care for having dementia) has influenced

your health care system movement?

a. How do you think it differs from that of those without dementia?

EXAMPLE OF A PROBING QUESTION BASED ON PREVIOUS INTERVIEWS

6. We would like to know a little be about broader community supports for people with dementia –

things that are outside of the health care system (e.g., attitudes, perceptions about dementia in the

community, transportation, etc)

a. What things outside of your health care do you think influence your experience with

dementia/caring for someone with dementia?

b. What do you think can be done to make the wider community more supportive of people

with dementia?

161

ENDING QUESTIONS

7. Is there anything else you think I should know?

8. Is there anything that you would like to ask me?
